# Attitudes and perceptions toward artificial intelligence among practicing rheumatologists in Iraq: a cross-sectional web-based survey

**DOI:** 10.3389/fmed.2026.1896672

**Published:** 2026-10-16

**Authors:** Mustafa Alhayali, Marwa Moayad, Nizar Abdulatif Jasim, Zaid Al-Zuhairi, Marwah Al-Mashhadani, Mustafa A. Shahrori, Rami Al-Khaffaf, Marafi Jammaa Ahmed, Enas Osama Hassan Omer, Aya Ahmed Shimal, Rafal Othman, Ahmed Dheyaa Al-Obaidi

**Affiliations:** 1 Department of Rheumatology, College of Medicine, Ibn Sina University of Medical and Pharmaceutical Sciences, Baghdad, Iraq; 2 College of Medicine, University of Baghdad, Baghdad, Iraq; 3 Department of Internal Medicine, Aldujail Hospital, Salah al-Din, Iraq; 4 Faculty of Medicine, An-Najah National University, Nablus, Palestine; 5 Department of Internal Medicine, Henry Ford St. John Hospital, Detroit, MI, United States; 6 Faculty of Medicine, University of Bahri, Khartoum, Sudan; 7 Albany Medical College, Albany, NY, United States

**Keywords:** AI, artificial intelligence, attitudes, Iraq, perceptions, physicians, rheumatology

## Abstract

**Background:**

Artificial intelligence is becoming a bigger part of healthcare, particularly in rheumatology, where clinicians must integrate complex clinical, laboratory, and imaging data. Physicians’ perceptions of AI may substantially influence the extent to which it is implemented responsibly in routine clinical practice.

**Objective:**

This study aimed to assess the attitudes of practicing rheumatologists in Iraq toward artificial intelligence and to examine their association with demographic and professional characteristics.

**Methods:**

A four-month web-based cross-sectional survey was conducted from October 2025 to January 2026. A questionnaire adapted from previous studies was refined through expert review and pilot testing before distribution. The questionnaire comprised demographic information, attitude statements measured on a five-point Likert scale, and items assessing general perceptions of artificial intelligence. Composite attitude scores were categorized using modified Bloom's cutoff criteria. Statistical analysis was performed using SPSS and R.

**Results:**

A total of 162 complete responses were collected. Most respondents were aged 35–44 years (42.0%), male (56.8%), and specialists (67.9%). Overall, 61.7% reported familiarity with AI and 69.1% had already used AI in rheumatology practice. Most participants 56.8% exhibited a poor attitude, 32.1% a moderate attitude, and 11.1% a good attitude toward AI. Bivariate analysis identified familiarity with AI (p=0.003) and involvement in research (p=0.009) as significantly associated with more favorable attitudes.

**Conclusion:**

Iraqi rheumatologists demonstrate cautious engagement with artificial intelligence, with the majority displaying poor-to-moderate overall attitude scores on the standardized composite scale. Greater familiarity with AI and research involvement were associated with more favorable attitudes, highlighting the importance of targeted digital literacy and specialty-specific education to support effective AI adoption in clinical practice.

## 1 Introduction

Artificial intelligence is making its mark on healthcare by helping with everything from clinical decisions and medical imaging to predicting patient outcomes and streamlining office workflows. With the rapid rise of machine learning and generative AI, there is growing enthusiasm for integrating these technologies into clinical practice. However, this transition also raises important concerns about transparency, bias, and the continued reliability of human clinical judgment (1–3).

In rheumatology, AI is particularly well-suited given the need to integrate diverse data types over extended periods. Emerging evidence suggests that these technologies may enable earlier disease diagnosis, improve patient stratification, and advance the shift toward personalized care in autoimmune diseases (4–7).

For AI to be effectively integrated into clinical practice, its success depends not only on technological performance but also on clinicians’ trust, understanding, and ability to evaluate its outputs critically. Previous studies indicate that, although many physicians are receptive to the concept of AI, they often report limited formal training and insufficient confidence to use these tools safely. For instance, research among Arab medical students has demonstrated high levels of interest alongside minimal structured education in AI. At the same time, other studies suggest that physicians’ attitudes are influenced more by practical experience with these technologies than by demographic factors such as age or background (8, 9).

These issues are particularly pressing in healthcare systems that are still updating their digital infrastructure. We know from broader research that whether a clinician actually uses AI depends more on how it fits into their daily routine and on whether they find it useful than on simply having the software available (10, 11).

Even though AI in rheumatology is a hot topic internationally, we lack data on how rheumatologists in Iraq and the surrounding region feel about it. Major organizations such as EULAR have pointed out that while big data and AI are growing rapidly, we need stronger regulations and much better clinician education (6, 12). This study was designed to bridge this gap by examining the attitudes and perceptions of Iraqi rheumatologists and assessing the factors that influence their views.

## 2 Methods

### 2.1 Study design and setting

A web-based cross-sectional survey was conducted over 4 months from October 2025 to January 2026 among practicing rheumatologists in Iraq.

### 2.2 Participant flow and sampling

The target population comprised practicing rheumatologists across Iraq. The survey invitation link was distributed electronically to approximately 225 eligible rheumatologists through national specialty networks and professional WhatsApp groups. A total of 180 physicians opened the survey link, of whom 166 initial participants commenced filling out the questionnaire. Following data validation and cleaning, 4 incomplete responses and entries from physicians younger than 25 years were excluded. The final sample included in the statistical analysis comprised 162 complete responses.

The participation rate among those who initiated the survey was 97.6% (162/166). The overall study response rate of 72.0% (162/225) was calculated based on the total estimated pool of eligible invited clinicians using the following formula:

$$\begin{array}{rl} & \mathrm{Response}\;\mathrm{Rate}\;(\text{\%})=\\ & \left(\frac{\mathrm{Number}\;\mathrm{of}\;\mathrm{Complete}\;\mathrm{Analyzed}\;\mathrm{Responses}\;(n=162)}{\mathrm{Total}\;\mathrm{Number}\;\mathrm{of}\;\mathrm{Eligible}\;\mathrm{Invited}\;\mathrm{Rheumatologists}\;(N=225)}\right)\\ & \times 100 \end{array}$$

### 2.3 Questionnaire development

The questionnaire consisted of 3 sections with multiple-choice questions: demographic information (6 items), attitudes toward AI (8 items), and perceptions of AI (4 items). The instrument was developed and adapted from previously published surveys assessing attitudes and perceptions toward AI in healthcare (8, 9). Content validity was established through expert review, and a pilot study was conducted to assess clarity and feasibility; minor modifications were made accordingly. The complete instrument used for data collection is available as Supplementary File S1.

### 2.4 Attitude scoring

The attitude-related responses initially comprised eight 5-point Likert items. Each item is scored from 1 to 5. Before computing a composite score, the scale was assessed for internal consistency and conceptual coherence. Although the overall reliability was acceptable (Cronbach's *α* = 0.84), item-level diagnostics indicated that two items did not measure the same underlying construct as the remaining attitude items. Therefore, as shown in Figure 1, the final attitude score is based on six items assessing the perceived impact of AI, the necessity of AI knowledge, workload reduction, diagnostic improvement, confidence in evaluating AI information, and AI replacing certain aspects of practice. The total attitude score ranges from 6 to 30, with higher scores indicating a more positive attitude toward AI. Attitude levels were categorized according to the modified Bloom's cut-off criteria as poor (<60% of the maximum score), moderate (60–79%), and good (≥80%).

**Figure 1:**
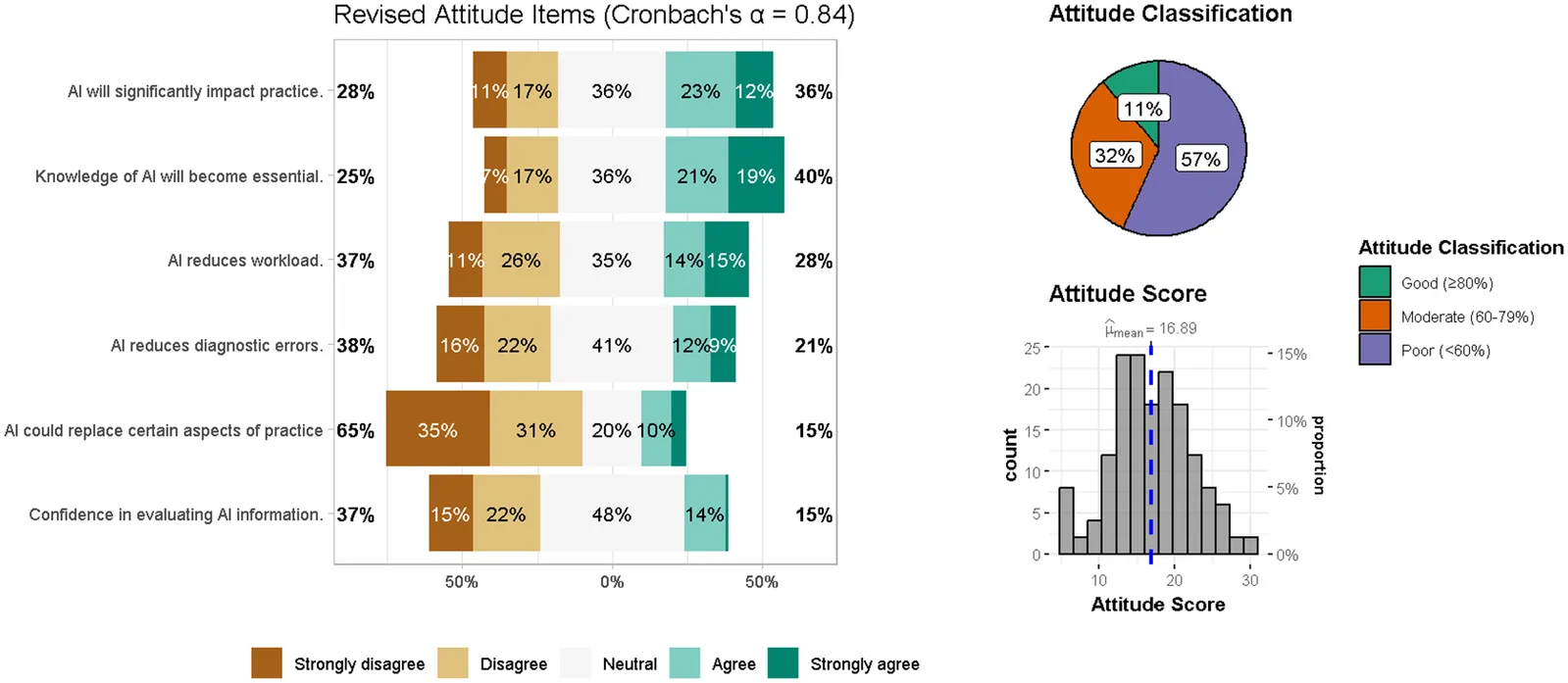
Revised 6-item attitude scale responses, overall attitude score distribution, and attitude classification.

### 2.5 Data collection

The questionnaire was distributed via WhatsApp groups to eligible participants and completed electronically. The survey collected information on demographic characteristics, familiarity with AI, prior use of AI in rheumatology practice, attitudes toward AI, and perceptions regarding training, verification practices, future impact, and concerns.

### 2.6 Ethical considerations

Approval was obtained from the Scientific Committee of the Rheumatology Department and the Scientific Committee of the College of Medicine, Ibn Sina University of Medical and Pharmaceutical Sciences. Online informed consent was obtained from all participants. Confidentiality was maintained throughout the study.

### 2.7 Statistical analysis

Data were analyzed using the Statistical Package for Social Sciences (SPSS) version 26. Categorical data were presented as frequencies and percentages. Inferential statistics were performed using R statistical software (Version 4.4.3). Chi-square test and Ordinal logistic regression, with adjusted odds ratios, were used to assess associations between participant characteristics and attitude score classification. A *p*-value of 0.05 or less was considered statistically significant.

## 3 Results

The response rate was 72%. Table 1 presents the socio-demographic characteristics of the respondents and their responses regarding AI. The majority of participants were between 35–44 years (42.0%), followed by those aged 25–34 years (32.1%), while smaller proportions were in the age groups 45–54 years (12.3%), 55–64 years (7.4%), and above 64 years (6.2%).

**Table 1:** Sociodemographic characteristics of participating rheumatologists and their responses regarding familiarity with and use of artificial intelligence.

Socio-demographic distribution of the participants and their answers regarding AI			
Variables		No.	%
Age	25–34	52	32.1
	35–44	68	42.0
	45–54	20	12.3
	55–64	12	7.4
	> 64	10	6.2
Gender	Male	92	56.8
	Female	70	43.2
Area of practice	Hospital	70	43.2
	Private clinic	4	2.5
	Academic	4	2.5
	Research	0	0.0
	More than one option	84	51.9
Current role	Trainee	52	32.1
	Specialist	110	67.9
Are you familiar with AI?	No	14	8.6
	Yes	100	61.7
	May be	48	29.6
Have you used AI in rheumatology practice?	No	50	30.9
	Yes	112	69.1
Total		162	100

Males represented a higher proportion of the study population (56.8%) compared to females (43.2%). With regard to area of practice, 43.2% worked in hospitals, whereas only 2.5% practiced exclusively in private clinics and another 2.5% in academic institutions only.

None of the respondents reported working exclusively in research, this is because of the absence of a dedicated research-focused specialty within rheumatology in Iraq, while the largest proportion (51.9%) indicated involvement in more than one practice area. In terms of professional role, specialists constituted the majority (67.9%), with trainees accounting for 32.1%.

Concerning knowledge of AI, most respondents were familiar with AI (61.7%), while 29.6% responded “maybe,” and only 8.6% reported no familiarity. Notably, 69.1% had already used AI in rheumatology practice, whereas 30.9% had not.

Figure 2 illustrates the distribution of respondents’ professional practice in the field. More than half of the respondents reported fewer than 15 years of practice, whereas the frequency steadily declined among those with longer careers. Only a small proportion of rheumatologists reported more than 20 years of practice, and very few exceeded 30 years.

**Figure 2:**
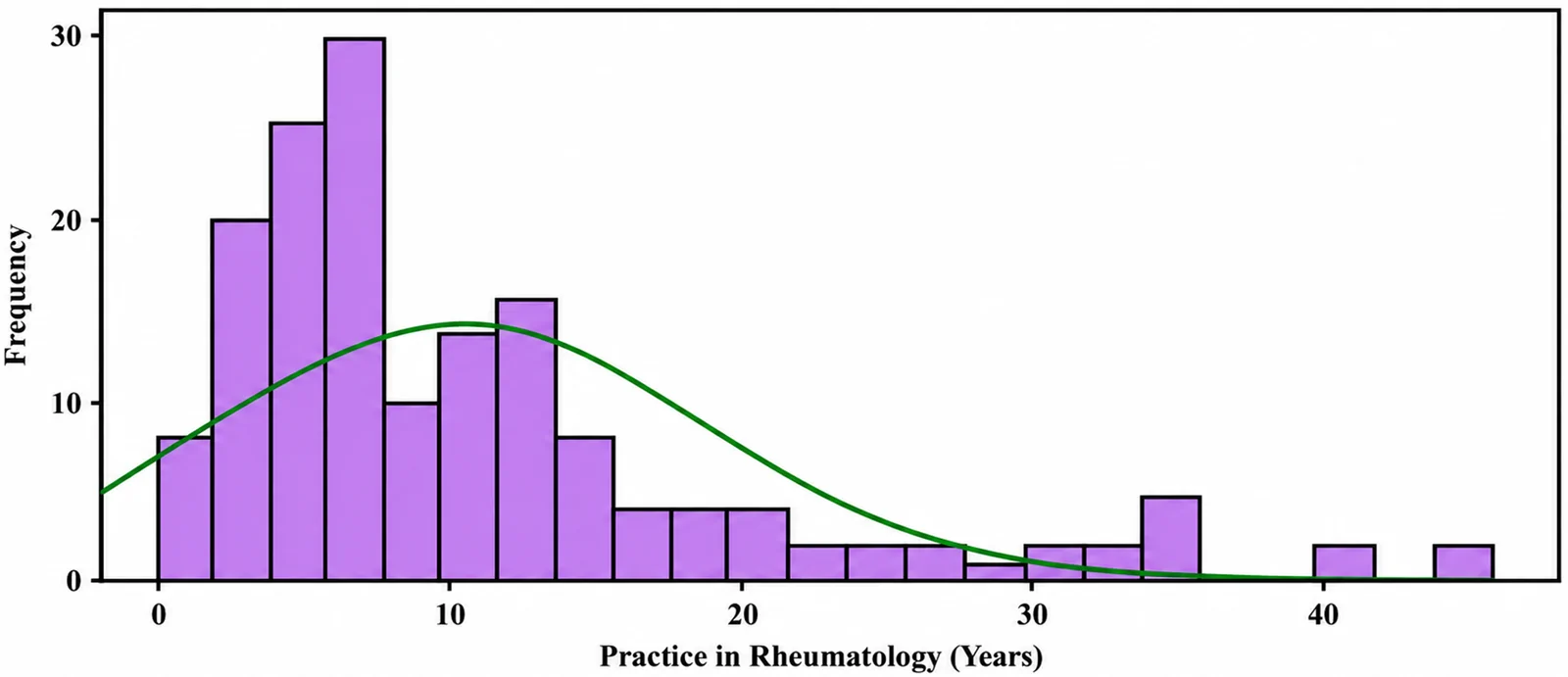
Distribution of participants according to years of experience in rheumatology practice.

Figure 3 shows that the majority of respondents were from Baghdad (42; 25.9%), followed by Erbil (22, 13.6%). Salahuddin and Kerkuk each contributed 12 respondents (7.4%). Najaf, Diyala, Sulaymaniyah, Basrah, and Babylon each accounted for 10 respondents (6.2%). Kerbala represented 6 respondents (3.7%), while Aldiwaniya and Ninevah each contributed 4 respondents (2.5%). Smaller proportions of respondents were recorded from Anbar, Maisan, Kut, Muthana, and Thi Qar, with 2 participants from each governorate (1.2%), while no respondents were recorded from Duhok.

**Figure 3:**
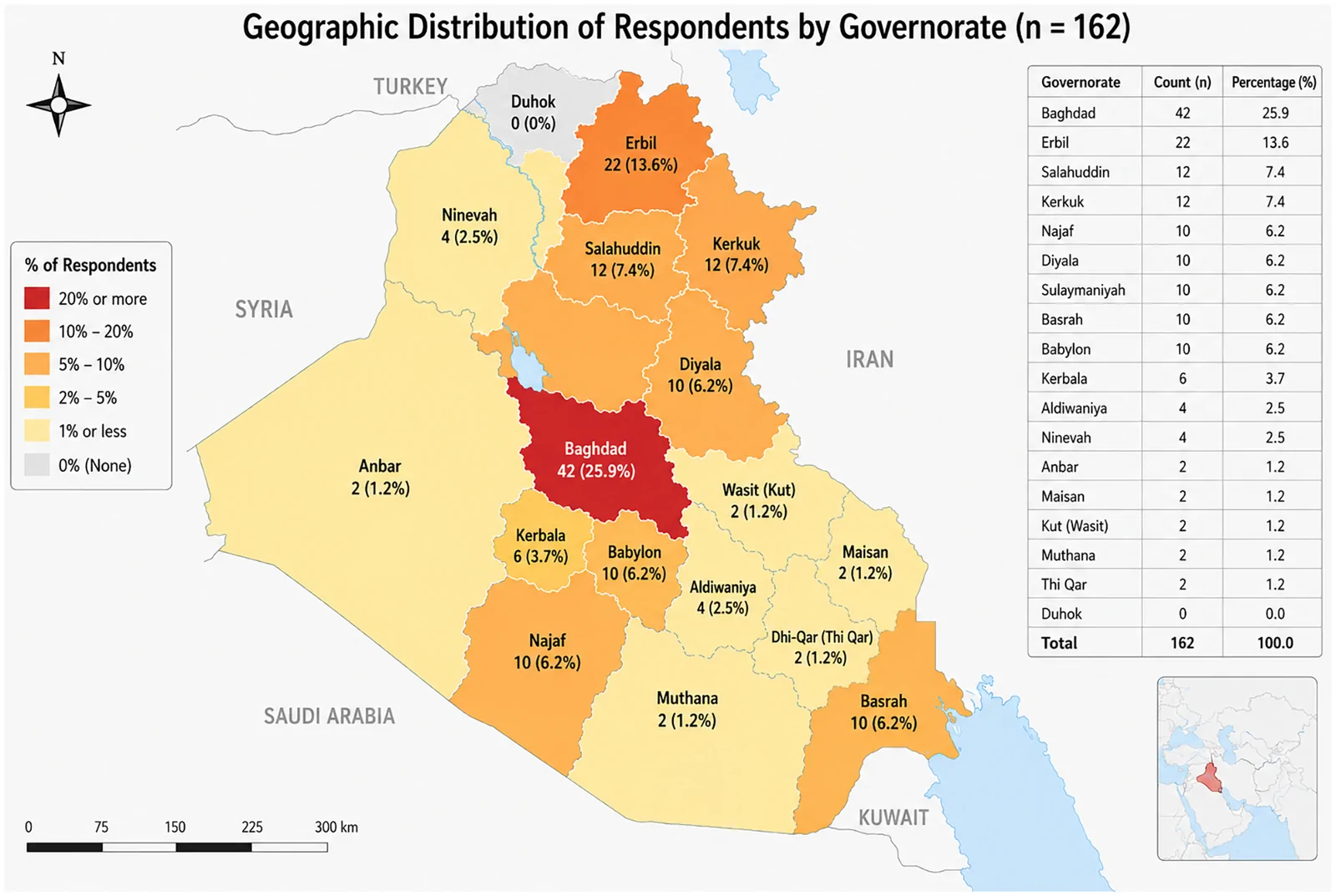
Geographic distribution of participating rheumatologists across Iraqi governorates.

Figure 4 demonstrates that the most widely used tool among participants was ChatGPT (OpenAI), reported by 74 respondents (46%). This was followed by Google Bard (Gemini) with 58 respondents (∼36%), and “None” with 18 participants (11%), indicating they had not used any AI tool. Smaller proportions were recorded for Grok (xAI) (8; 5%), Anthropic Claude (2; 1%), and Medical-specific AI tools (2; 1%), while no participants reported the use of DeepSeek, Microsoft Copilot, or Perplexity AI.

**Figure 4:**
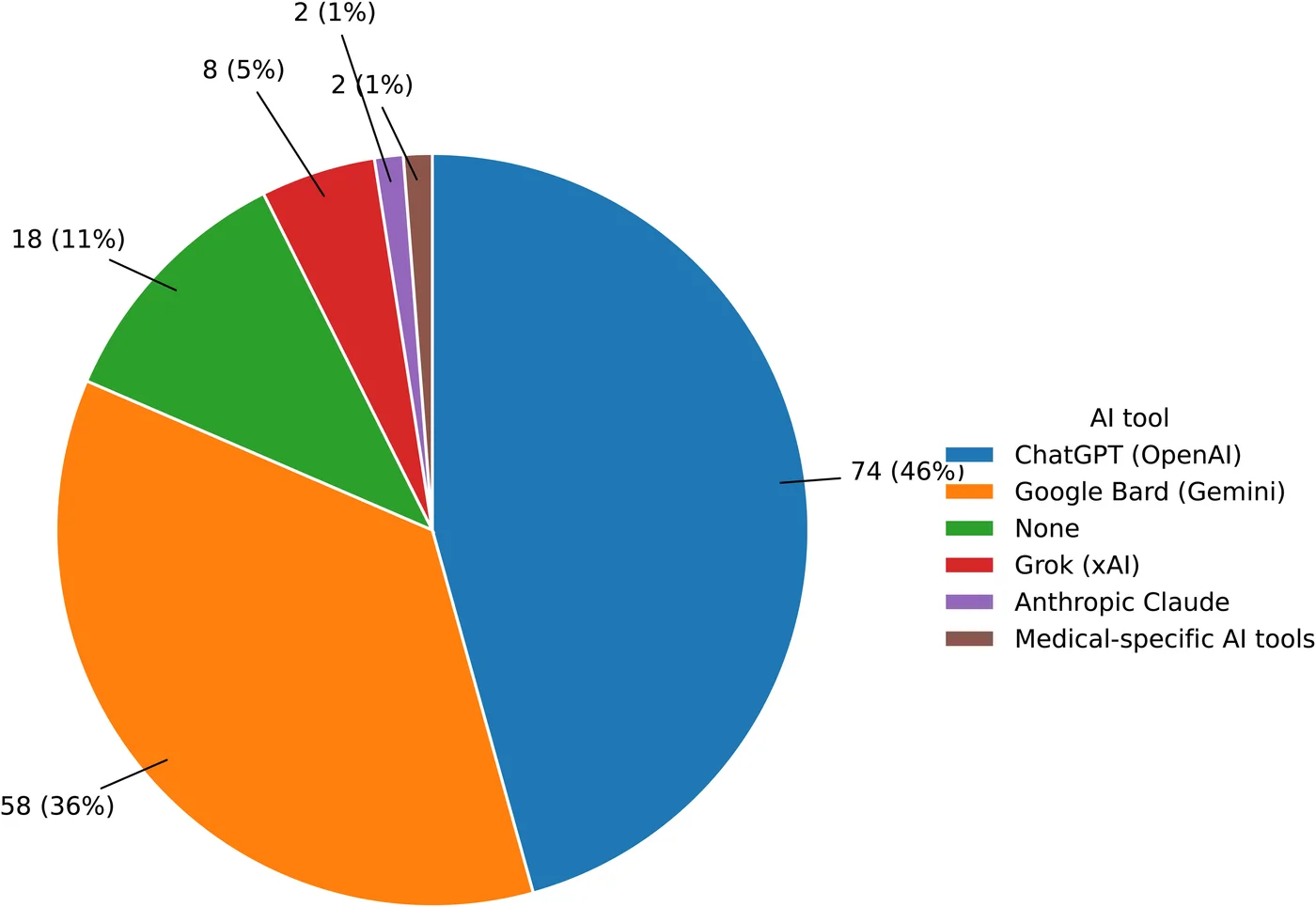
Artificial intelligence tools reported by study participants.

Table 2 illustrates respondents’ attitudes toward AI in rheumatology. The largest proportion of respondents remained neutral when asked whether AI will significantly impact rheumatology practice (35.8%) and whether knowledge of AI will become essential for rheumatologists (35.8%).

**Table 2:** Distribution of participant responses to attitude statements regarding artificial intelligence in rheumatology practice.

Questions on participants’ attitudes toward AI			
Questions		No.	%
AI will significantly impact rheumatology practice	Strongly disagree	18	11.1
	Disagree	28	17.3
	Neutral	58	35.8
	Agree	38	23.5
	Strongly agree	20	12.3
Knowledge of AI will become essential for rheumatologists	Strongly disagree	12	7.4
	Disagree	28	17.3
	Neutral	58	35.8
	Agree	34	21.0
	Strongly agree	30	18.5
AI has the potential to reduce workload for rheumatologists	Strongly disagree	18	11.1
	Disagree	42	25.9
	Neutral	56	34.6
	Agree	22	13.6
	Strongly agree	24	14.8
AI could reduce diagnostic errors in complex autoimmune cases	Strongly disagree	26	16.0
	Disagree	36	22.2
	Neutral	66	40.7
	Agree	20	12.3
	Strongly agree	14	8.6
I am concerned about the ethical implications of AI use in medicine.	Strongly agree	24	14.8
	Agree	40	24.7
	Neutral	48	29.6
	Disagree	32	19.8
	Strongly disagree	18	11.1
AI could replace certain aspects of rheumatology practice	Strongly agree	56	34.6
	Agree	50	30.9
	Neutral	32	19.8
	Disagree	16	9.9
	Strongly disagree	8	4.9
How confident are you in evaluating AI information	Strongly disagree	24	14.8
	Disagree	36	22.2
	Neutral	78	48.1
	Agree	22	13.6
	Strongly agree	2	1.2
Patients should be informed when AI is involved in their diagnosis or treatment	Strongly disagree	60	37.0
	Disagree	22	13.6
	Neutral	44	27.2
	Agree	8	4.9
	Strongly agree	28	17.3
Total		162	100

Similarly, neutrality was also the most common response regarding AI's potential to reduce workload (34.6%) and its ability to reduce diagnostic errors in complex autoimmune cases (40.7%). When considering ethical concerns, the highest proportion (29.6%) also reported a neutral stance.

In contrast, regarding the statement that AI will replace certain aspects of rheumatology practice, the majority strongly agreed (34.6%). Regarding confidence in evaluating AI information, nearly half of the participants (48.1%) were neutral, indicating uncertainty in their ability to critically assess AI information.

Finally, on whether patients should be informed when AI is involved in diagnosis or treatment, the largest proportion of participants strongly disagreed (37.0%).

The 6-item attitude scale and classification were visualized in Figure 1. Among 162 participants (poor attitude, *n* = 92; moderate, *n* = 52; good, *n* = 18), bivariate analysis revealed significant associations between attitude classification and both research involvement (*p* = 0.009) and AI familiarity (*p* = 0.003). Participants engaged in research, and those reporting familiarity with AI were disproportionately represented in the moderate/good attitude categories. Prior AI use in practice showed a similar trend (63.0% vs. 88.9%, poor vs. good, *p* = 0.072). However, in the multivariable ordinal logistic regression, neither research involvement (OR 2.09, 95% CI 0.86–5.11, *p* = 0.10) nor AI familiarity (OR 4.39, 95% CI 0.79–36.3, *p* = 0.12) remained significant after adjustment, despite point estimates in the expected direction. The single significant predictor was age >64 (OR 0.10, 95% CI 0.01–0.64, *p* = 0.022). But, notably, zero participants aged >64 fell into the good attitude category, indicating the odds ratio derives from a near-empty cell (Tables 3, 4).

**Table 3:** Association between sociodemographic characteristics, artificial intelligence experience, and attitude classification among participants.

Characteristic	Poor (<60%) *N* = 92^a^	Moderate (60–79%) *N* = 52^a^	Good (≥80%) *N* = 18^a^	*p*-value^b^
Age				0.11
25–34	24 (26.1%)	24 (46.2%)	4 (22.2%)	
35–44	40 (43.5%)	18 (34.6%)	10 (55.6%)	
45–54	10 (10.9%)	6 (11.5%)	4 (22.2%)	
55–64	10 (10.9%)	2 (3.8%)	0 (0.0%)	
>64	8 (8.7%)	2 (3.8%)	0 (0.0%)	
Sex				0.2
Male	50 (54.3%)	34 (65.4%)	8 (44.4%)	
Female	42 (45.7%)	18 (34.6%)	10 (55.6%)	
Research	8 (8.7%)	14 (26.9%)	4 (22.2%)	0.009
Current role				0.4
Specialist	64 (69.6%)	32 (61.5%)	14 (77.8%)	
Trainee	28 (30.4%)	20 (38.5%)	4 (22.2%)	
Are you familiar with Artificial Intelligence?				0.003
No	12 (13.0%)	2 (3.8%)	0 (0.0%)	
Yes	46 (50.0%)	42 (80.8%)	12 (66.7%)	
Maybe	34 (37.0%)	8 (15.4%)	6 (33.3%)	
Have you used AI in Rheumatology practice?	58 (63.0%)	38 (73.1%)	16 (88.9%)	0.072

a *n* (%).

b Fisher's exact test; Pearson's Chi-squared test.

**Table 4:** Ordinal logistic regression predicting positive attitude toward AI.

Characteristic	OR	95% CI	*p*-value
Age			
25–34	–	–	
35–44	0.32	0.09, 1.03	0.063
45–54	0.47	0.11, 2.04	0.3
55–64	0.19	0.02, 1.30	0.11
>64	0.10	0.01, 0.64	0.022
Sex			
Male	–	–	
Female	0.88	0.44, 1.75	0.7
Research			
No	–	–	
Yes	2.09	0.86, 5.11	0.10
Current role			
Specialist	–	–	
Trainee	0.38	0.10, 1.27	0.13
Are you familiar with Artificial Intelligence?			
No	–	–	
Yes	4.39	0.79, 36.3	0.12
Maybe	2.11	0.41, 16.4	0.4
Have you used AI in Rheumatology practice?			
No	–	–	
Yes	1.18	0.47, 3.01	0.7

CI, confidence interval; OR, odds ratio.

Table 5 outlines respondents’ perceptions regarding AI in rheumatology. When asked how often they verify AI answers with external sources, the largest proportion responded “sometimes” (46.9%), indicating a moderate level of caution.

**Table 5:** Participants’ perceptions regarding artificial intelligence use, training requirements, anticipated impact, and concerns in rheumatology practice.

Questions on participants’ perception toward AI			
Questions		No.	%
How often do you verify AI answers with external sources?	Never	16	9.9
	Rarely	14	8.6
	Sometimes	76	46.9
	Often	40	24.7
	Always	16	9.9
Do you believe AI training in rheumatology should be?	Mandatory	12	7.4
	Optional soft skills	114	70.4
	Not necessary	16	9.9
	I don't know	20	12.3
Which areas do you think AI will most impact?	Clinical decision making	10	6.2
	Medical education	20	12.3
	Research	18	11.1
	Patient communication	2	1.2
	More than one option	112	69.1
What concerns do you have?	Over reliance on technology	8	4.9
	Loss of traditional clinical skills	12	7.4
	Need to update technical skills constantly	12	7.4
	Job security	2	1.2
	Quality of patient care	2	1.2
	More than one option	126	77.8
Total		162	100

Regarding AI training, most participants (70.4%) believed it should be offered optional soft skills, while only a minority considered it mandatory or unnecessary. Concerning the areas of greatest impact, the majority (69.1%) selected “more than one option”, reflecting the view that AI will influence multiple aspects of rheumatology, such as clinical decision-making, medical education, research, and patient communication, rather than a single domain.

Similarly, when asked about their concerns, the overwhelming majority (77.8%) also chose “more than one option”, suggesting that participants perceive several overlapping risks, such as overreliance, skill loss, or patient care quality, rather than a single dominant issue.

Table 6 presents a comparative analysis of Iraqi rheumatologists’ perspectives on artificial intelligence against findings from recent international studies on perceptions of clinical AI.

**Table 6:** Comparison of Iraqi rheumatologists’ perspectives on artificial intelligence with findings from similar international studies.

Metric/Domain	Current Study (Iraq, 2026)	Mahadevan et al. (India, 2025)	Heinrichs et al. (Germany, 2025)
Population	*N* = 162	*N* = 173	*N* = 498
Cohort Profile	Practicing Rheumatologists	Rheumatology Consultants and Residents	Hospital and Private Practice Physicians
General AI Familiarity/Awareness	61.7% reported clear familiarity	98.3% general awareness (though only 54.3% understood specific technical sub-terms)	Highly variable; correlated strongly with prior professional or personal use
Current Clinical/Tool Use	69.1% have used AI tools in rheumatology practice (predominantly general LLMs like ChatGPT at 46.0%)	33.5% regularly use AI tools in clinical practice	Limited routine deployment; primarily used by those in active research settings
Primary Core Sentiment	High levels of neutrality across Likert items (e.g., 35.8%–48.1% neutral); overall 56.8% poor, 32.1% moderate, 11.1% good attitude scores	High belief in clinical utility (81.5% state it can enhance care) but heavily constrained by data concerns	Generally positive; defined by High Enthusiasm paired with practical skepticism
Top Concerns/ Barriers	Overlapping concerns (77.8% selected multiple options): overreliance, loss of clinical skills, technical updates, data quality, and lack of clear regulatory frameworks	Patient data security (64.2%), ethical implications (25.4%), and lack of formal training	Usability hurdles, algorithmic transparency, liability, and shifts in doctor-patient dynamics
Key Significant Predictor	Bivariate: AI familiarity (p=0.003) and research involvement (p=0.009) Multivariable: Age >64 (OR=0.10, p=0.022; AI familiarity p=0.12, research p=0.10)	Prior specialized or hands-on technology training	Familiarity and direct research engagement (*p* < 0.001)

## 4 Discussion

This study provides a focused insight into how Iraqi rheumatologists currently perceive artificial intelligence. Overall, most participants demonstrated a poor-to-moderate attitude toward AI based on the standardized composite scale (56.8% poor, 32.1% moderate, and 11.1% good), with individual item responses reflecting high levels of neutrality. Notably, a substantial proportion reported familiarity with AI (61.7%), including its use in clinical practice (69.1%). These findings suggest that while AI has begun to integrate into the professional environment of Iraqi rheumatologists, its adoption is characterized by cautious engagement rather than unreserved enthusiasm.

That neutral stance is quite telling. It does not mean they are rejecting technology. Instead, it looks like a classic transitional phase: these clinicians can clearly see that AI will be important, but they are not yet sure whether it is reliable, ethical, or genuinely useful for day-to-day patient care. This perfectly echoes what we see in the wider literature. Doctors tend to sit right in the middle, optimistic about what AI could do, but worry about its current flaws, especially since formal training and clear guidelines are still largely a work in progress (1, 2, 9, 10).

Our results also line up with previous research. For example, Allam et al. found that Arab medical students understood AI's importance even without much formal training (8). Heinrichs et al. noted that a doctor's attitude toward AI relies heavily on their experience with it rather than on demographic traits (9). In our bivariate analysis, familiarity with AI (p=0.003) and research involvement (p=0.009) were significantly associated with more favorable attitude scores. However, after adjusting for potential confounders in multivariable ordinal logistic regression, familiarity (p=0.12) and research involvement (p=0.10) were no longer statistically significant, leaving age >64 as the sole significant predictor (OR=0.10,p=0.022).

This is a key finding. Although bivariate associations highlight the role of exposure, physician hesitation does not necessarily reflect resistance to innovation, but rather underlying uncertainty, gaps in structured training, and limited technological literacy. Our findings also present an intriguing contrast to tech-intensive specialties outside of rheumatology. For instance, a nationwide study of Indian radiologists by Goyal et al. revealed a highly active landscape where over 82% recognized AI's immediate impact on diagnostic accuracy, despite persistent anxieties regarding job displacement. This suggests that fields relying on discrete imaging face immediate, high-stakes exposure, whereas systemic specialties requiring longitudinal clinical data synthesis approach AI integration with greater observational caution (13).

Regarding training, most surveyed rheumatologists felt that AI education should be offered as an optional, complementary skill. This reflects a pragmatic perspective: while AI is clearly relevant, it may not yet be sufficiently standardized or mature to warrant mandatory inclusion for all practitioners. However, the expanding body of research in rheumatology suggests that a foundational understanding of AI is rapidly becoming essential. Indeed, its applications now extend well beyond image interpretation to include disease classification, outcome prediction, longitudinal monitoring, and modelling patient responses to therapy (4–7, 12).

It is also important to examine the specific tools being used. Interestingly, general-purpose large language models, such as ChatGPT, appear to be more widely utilized than specialized medical AI systems. This likely reflects their accessibility, low barrier to entry, and publicity.

However, this trend carries inherent risks. General-purpose LLMs lack the validated boundaries of specialty-specific systems and are prone to “hallucinations”. This introduces severe vulnerabilities regarding patient data privacy, security breaches and the lack of a standardised clinical audit trail (14). This heavy reliance highlights an urgent need to develop secure, medically sandboxed local interfaces that can bridge the gap between general accessibility and clinical safety.

Furthermore, although Iraqi rheumatologists demonstrated a generally receptive attitude toward AI, general-purpose AI models may generate inaccurate or unverifiable information. In our study, participants reported only intermittent verification of AI-generated outputs, which could increase the risk of inaccurate diagnostic suggestions if used without appropriate physician oversight. This aligns with ongoing discussions within the medical community emphasizing the need for responsible use of generative AI, particularly in complex and nuanced fields such as rheumatology (15, 16).

Ethical concerns were a major issue for our cohort. Many participants were worried about the moral implications of using AI in rheumatology, and they were completely split on whether patients need to be told when AI helps with their diagnosis or treatment. Notably, the largest proportion of participants strongly disagreed with mandatory patient disclosure (37.0%). This is significant, especially since international guidelines are pushing hard for transparency, human oversight, accountability, and explainability as the core pillars of responsible healthcare AI (2, 16).

Several factors may explain this observation. Culturally, medical practice in the Middle East historically leans toward a paternalistic model, where physicians assume the full burden of complex technical choices to protect patients from unnecessary anxiety and preserve institutional trust. These classic ethical dynamics balance absolute transparency against perceived patient psychological vulnerability (17). From a healthcare-system standpoint, the current absence of explicit, localized legal frameworks or statutes governing clinical AI medical liability in Iraq causes physicians to view these technologies as internal diagnostic aids—comparable to automated laboratory calculators or standard electronic imaging software—rather than independent entities requiring explicit informed consent.

Furthermore, a methodological misunderstanding of the survey question cannot be ruled out; respondents may have assumed the item referred to routine back-end administrative or imaging optimization tools rather than autonomous diagnostic systems, a common issue noted in broader physician AI surveys (18).

In our study, some participants may view disclosure of AI use as essential for respecting patient autonomy and informed consent. In contrast, others may worry that such disclosure could reduce patient confidence in physicians. The lack of clear regulatory and ethical frameworks for AI implementation in healthcare in Iraq may further contribute to these concerns. These findings highlight the importance of developing transparent governance policies, strengthening physician education about AI, and establishing ethical guidelines to support safe and trustworthy integration of AI into clinical practice.

All this fits right in with the broader rheumatology literature. EULAR-linked studies have shown that AI and big data are expanding rapidly in the field of rheumatic and musculoskeletal diseases, and we desperately need proper governance, rigorous methods, and doctors who understand the technology (6, 12). Other recent surveys have identified the same knowledge gaps, highlighting the need for structured AI education tailored specifically for rheumatologists (18).

From a practical standpoint, these findings highlight several clear priorities. First, educational initiatives for rheumatologists must transition from basic awareness to structured, curriculum-based strategies. Residency and fellowship programs should integrate formal digital literacy modules that teach the mechanics of clinical prompt engineering, data privacy compliance, and the identification of algorithmic hallucinations. Implementing practical, case-based simulation workshops—where trainees are required to cross-examine AI-generated differentials against established diagnostic criteria (such as EULAR or ACR guidelines)—will build the critical appraisal and verification skills required for safe adoption. Second, any initiative to integrate AI into clinical practice must account for real-world constraints, including workflow compatibility, institutional trust, and broader governance frameworks (10, 11). Third, local professional bodies and academic institutions must design targeted continuous medical education (CME) pathways and context-specific guidelines to provide practicing clinicians with ongoing training on the evolving ethical boundaries of generative AI within the specialty.

Despite providing valuable insights, this study has limitations. The cross-sectional design precludes definitive conclusions regarding causality. Additionally, sampling via professional WhatsApp groups introduces potential selection bias, as this approach inherently favors younger and more technologically engaged clinicians. This demographic tilt may limit the external validity of our findings and could artificially elevate the reported rates of AI familiarity and tool usage. Furthermore, the findings reflect self-reported data rather than an objective assessment of clinical proficiency. Nevertheless, these data establish a critical baseline for a historically underrepresented region. This research identifies key priorities for future inquiry, including multicenter longitudinal studies to evaluate the evolution of clinician attitudes as AI integration becomes standard in clinical practice.

## 5 Conclusion

Iraqi rheumatologists demonstrate cautious engagement with artificial intelligence, with the majority displaying poor-to-moderate overall attitude scores on the standardized composite scale. While greater familiarity with AI and active research involvement were associated with more favorable attitudes in preliminary comparisons, multivariable analysis showed that demographic factors like older age were key predictors of lower attitude scores. These findings highlight the importance of targeted digital literacy, clear regulatory governance, and specialty-specific education to support effective, safe, and responsible AI adoption in clinical practice.

## Data Availability

The raw data supporting the conclusions of this article will be made available by the authors, without undue reservation.

## Supplementary material

Supplementary File S1 Web-Based Survey Questionnaire used for assessing attitudes and perceptions toward artificial intelligence among practicing rheumatologists in Iraq.
