# Supplementary material for: Attitudes and perceptions toward artificial intelligence among practicing rheumatologists in Iraq: a cross-sectional web-based survey
Source: Front Med (Lausanne). 2026 Oct 16;13:1896672. doi: 10.3389/fmed.2026.1896672 (PMC13719341; doi:10.3389/fmed.2026.1896672)
Supplement: Supplementary file 1 [file Datasheet1.pdf]

AI

Please answer all questions

* Indicates required question

1. Governorate *

Mark only one oval.

- Baghdad
- Basrah
- Ninevah
- Erbil
- Sulaimaniya
- Duhok
- Salahuddin
- Anbar
- Najaf
- Diyala
- Kut
- Kerbala
- Muthana
- Maisan
- Thi Qar
- Babylon
- Halabja
- Aldiwaniya
- Kerkuk

2. Age *

Mark only one oval.

- 25-34
- 35-44
- 45-54
- 55-64
- >64

3. Sex *

Mark only one oval.

- Male
- Female

4. Practice in Rheumatology (Years) *

____

5. Area of Practice (Tick all that apply) *

Check all that apply.

- [ ] Hospital
- [ ] Private Clinic
- [ ] Academic
- [ ] Research

6. Current role *

Mark only one oval.

- Trainee
- Specialist

7. Are you familiar with Artificial Intelligence? *

Mark only one oval.

- Yes
- No
- Maybe

8. Have you used AI in Rheumatology practice? *

Mark only one oval.

- Yes
- No

9. Which Large Language Models did you use? (Tick all that apply) *

Check all that apply.

- [ ] ChatGPT (Open AI)
- [ ] Google Bard (Gemini)
- [ ] DeepSeek
- [ ] Grok(xAI)
- [ ] Microsoft Copilot
- [ ] Anthropic Claude
- [ ] Medical-specific AI tools
- [ ] Perplexity AI
- [ ] Others
- [ ] None

10. AI will significantly impact Rheumatology practice *

1	2	3	4	5
☆	☆	☆	☆	☆

11. Knowledge of AI will become essential for rheumatologists *

1	2	3	4	5
☆	☆	☆	☆	☆

12. AI has the potential to reduce workload for rheumatologists *

1	2	3	4	5
☆	☆	☆	☆	☆

13. AI could reduce diagnostic errors in complex autoimmune cases *

1	2	3	4	5
☆	☆	☆	☆	☆

14. I am concerned about the ethical implications of AI use in medicine. *

1	2	3	4	5
☆	☆	☆	☆	☆

15. AI could replace certain aspects of Rheumatology practice *

1	2	3	4	5
☆	☆	☆	☆	☆

16. How confident are you in evaluating AI information *

1	2	3	4	5
☆	☆	☆	☆	☆

17. Patients should be informed when AI is involved in their diagnosis or treatment *

1	2	3	4	5
☆	☆	☆	☆	☆

18. How often do you verify AI answers with external sources *

Mark only one oval.

- Never
- Rarely
- Sometimes
- Often
- Always

19. Do you believe AI training in rheumatology should be *

Mark only one oval.

- Mandatory
- Optional soft skills
- Not necessary
- I don't know

20. Which areas do you think AI will most impact (Tick all that apply) *

Check all that apply.

- [ ] Clinical decision making
- [ ] Medical education
- [ ] Research
- [ ] Patient communication

21. What concerns do you have (Tick all that apply) *

Check all that apply.

- [ ] Over reliance on technology
- [ ] Loss of traditional clinical skills
- [ ] Need to update technical skills constantly
- [ ] Job security
- [ ] Quality of patient care
- [ ] Other (please specify)

22. What barriers do you perceive to implementing AI in rheumatology practice in Iraq? (optional)

____

This content is neither created nor endorsed by Google.

Google Forms
